# Effects of environmental enrichment and sexual dimorphism on the expression of cerebellar receptors in C57BL/6 and BTBR + Itpr3tf/J mice

**DOI:** 10.1186/s13104-022-06062-8

**Published:** 2022-05-13

**Authors:** Daniela Monje-Reyna, Jorge Manzo Denes, Fidel Santamaria

**Affiliations:** 1 Department of Neuroscience, Developmental and Regenerative Biology, University of Texas at San Antonio, San Antonio, TX 78249 US; 2 Brain Research Institute, Veracruzana University, Xalapa, Veracruz México

**Keywords:** Synaptic receptors, Excitability, Behavior, Plasticity, Multi-photon imaging, Autism spectrum disorders

## Abstract

**Objective:**

Environmental enrichment is used to treat social, communication, and behavioral deficits and is known to modify the expression of synaptic receptors. We compared the effects of environmental enrichment in the expression of glutamate and endocannabinoid receptors, which are widely expressed in the cerebellar cortex. These two receptors interact to regulate neuronal function and their dysregulation is associated with behavioral changes. We used BTBR + Itpr3tf/J mice, a strain that models behavioral disorders, and C57BL/6 mice for comparison. We studied the effects of genetic background, sex, environmental conditions, and layer of the cerebellar cortex on the expression of each receptor.

**Results:**

The influence of genetic background and environmental enrichment had the same pattern on glutamate and endocannabinoid receptors in males. In contrast, in females, the effect of environmental enrichment and genetic background were different than the ones obtained for males and were also different between the glutamate and endocannabinoid receptors. Furthermore, an analysis of both receptors from tissue obtained from the same animals show that their expression is correlated in males, but not in females. Our results suggest that environmental enrichment has a receptor dependent and sexual dimorphic effect on the molecular expression of different receptors in the cerebellar cortex.

**Supplementary Information:**

The online version contains supplementary material available at 10.1186/s13104-022-06062-8.

## Introduction

The BTBR + Itpr3tf/J (BTBR) mouse model is widely used in studies related to social and communication deficits, and repetitive behaviors [1–3]. BTBR mice exposed to environmental enrichment show decreases in repetitive behaviors and anxiety [4, 5], and show an increase in social affiliation [6]. In separate studies at the molecular level, changes in the expression of NMDAR1s [7, 8] and CB1Rs [9] are modulated by environmental enrichment. Interactions between NMDAR1s and CB1Rs, contribute to regulate neuronal function [10, 11], including in the cerebellum [12] where they are widely expressed in the cerebellar cortex [13, 14]. For these reasons, we quantified the expression of NMDAR1s and CB1Rs in the cerebellar cortex. In particular, we measured these changes in lobule VII because changes in the structure and physiology of this area correlate with abnormal behaviors such as compulsive rituals, stereotypical performance, and difficulty to understand social cues [15], which are replicated in the abnormal behavioral phenotype of the BTBR strain [16, 17]. The BTBR and C57 groups of each sex were exposed to a standard or enriched environmental conditions. Our results suggest that environmental enrichment has a receptor dependent and sexual dimorphic effect in the cerebellar cortex.

## Main text

### Methods

#### Animal procedures

We bred a new colony for 4 months of C57 (stock #000664) and BTBR (stock #002282) mice (Jackson Laboratories, Farmington, CT) under the protocol MU113, approved by the UTSA Institutional Animal Care and Use Committee (IACUC). Animals were maintained on a 12 h light/dark-cycle with constant access to food and water. After weaning, animals were separated by sex and housed with their litter in standard caging (26 cm width × 16 cm long x 16 cm deep) until P75. Reaching this age, animals from the same litter were randomly selected and separated in those that stayed in the standard environment (standard caging all the time) and those that experienced the environmental enrichment protocol.

The environmental enrichment arena was 90 cm width × 40 cm long × 33 cm deep and contained dust free bedding (Sophresh Natural Aspen) and toys. The toys were balls, cubes, and pyramids of different sizes, shapes, colors, and textures (solid, hollow, furry); there was also a running wheel, and tunnels. The position of all the toys was changed every day. Animals were placed in the arena 1 h a day for 20 days during the second four hours of the light part of their light cycle. Animals were returned to their standard cages after each exposure to environmental enrichment. At the end of the 20 days period the standard and environmental enrichment animals were euthanized. We anesthetized mice with 4 mL isoflurane in an evaporation chamber and kept them in deep anesthesia using the nose cone method. Animals were transcardially perfused with saline followed by 4% paraformaldehyde (0.2 M phosphate buffer, pH 7.4).

#### Imaging and data analysis

**Fig. 1:**
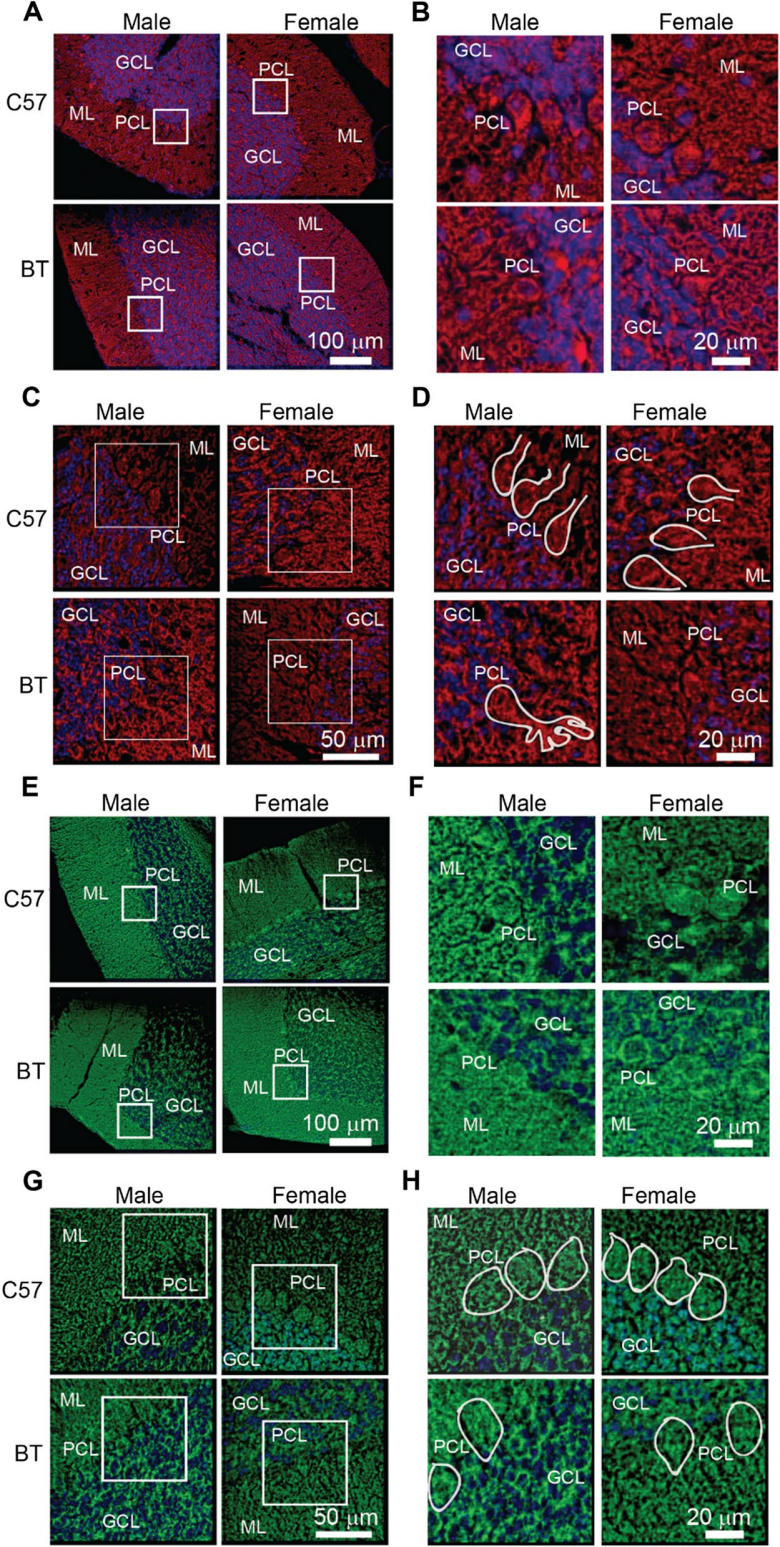
Expression of NMDAR1 and CB1R in the cerebellar cortex of C57BL/6 (C57) and BTBR + Itpr3tf/J (BT) mice raised in standard environments. **A** Fluorescent signal of NMDAR1 (red). **B** Images corresponding to the squares in **A**. **C** Images obtained from averaging the fluorescence of a Z-stack of 10 images separated by 1 µm. **D** images corresponding to the squares in **C**. The contours emphasize the shape of Purkinje cell somas and dendrites. **E–H** As in **A–F** for the expression of CB1 receptors (green) **H**. The contours emphasize the CB1R presynaptic terminals around Purkinje cell soma. ML, Molecular cell layer, PCL, Purkinje cell layer; GCL, Granule cell layer. All slices were stained with DAPI and identically processed (blue)

A detailed description of the immunofluorescence protocol is in the Additional file . We visualized expression of NMDAR1, CB1R, and DAPI with a multi-photon microscope (Bruker Ultima, Madison, WI). All images were collected with a 20 × objective. The wavelength of the tunable laser (DeepSee 690–1300 nm, Spectr Physics) to excite at the same time DAPI-NMDAR1 was 1120 nm and for DAPI-CB1R was 1078 nm. Fluorescent signals were separated by a dichroic cube and detected by photo multipliers. In all cases the scanning laser spent 2.0 µs in each coordinate of the image. Images for Fig. 1 were background corrected, thresholded, contrast enhanced, and convolved with a Gaussian filter to enhance morphological features. All images used for statistical analyses were only background corrected.

We selected ROIs for the molecular, Purkinje, and granular layers for each receptor in every slice. In the molecular and granular layers, the ROIs were polygons with and average area of 13,072 μm^2^ with a rage from 1718 to 45,365 μm^2^ for the granular and 7098 μm^2^ with a range of 1273–22,125 μm^2^ for the molecular layer. The ROI for the Purkinje cells were squares of 19.7 μm side. We collected, in average, 9.5 Purkinje cell somas per slice with a range from 5 to 15 (see Additional file 1: Figure S1 for an example of ROIs). The ROIs and background correction areas were identical for the receptor and DAPI images. In each ROI we computed a mask of the pixels that contained fluorescent information and used it to calculate the average value of the signal. We then calculated the ratio of NMDAR1/DAPI and CB1R/DAPI. Other groups have successfully used this technique to image brain slices and organoids [18].

For each animal we collected 3 images. The slices were selected based on a clear display of DAPI staining. Each experimental group consisted of 3 animals. No data points were excluded. Confounders were not controlled (such as animal/cage location). Images were analyzed blindly. We used a sample size and power test (*sampsizepwr* function in Matlab) to calculate the minimum number of animals necessary to have a statistical power of 80%. Based on our experimental measurements, the average standard deviation in each experimental group was 0.54. The number of animals required to have at least a difference of 2 in the expression of the receptors with this standard deviation was 3. We applied a Lilliefors test for all the data and then performed ANOVA and post-hoc tests as described in the results. In all cases we set significance at p ˂ 0.05. We also estimated the effect size of the ANOVA test using the value of ${\eta }^{2}=\frac{Sum Squares Effect}{Sum Squares Effect+SumSquares Error}$; for post hoc analyses we used Cohens’ d, $d=\frac{Mean group 1-Mean Group 2}{Population Standard Deviation}$ [19]. All the analyses were performed using custom MATLAB scripts (Natick, MA).

## Results

We found NMDAR1s present throughout the cerebellar cortex. NMDAR1s in Purkinje cell somas, dendrites of the molecular layer, and granular cell layer (Fig. 1A–D). We found CB1Rs expression on pre-synaptic terminals around Purkinje cell somas, consistent with their presence in the pinceau (Fig. 1E–H). There was also diffuse expression in the molecular layer, corresponding to parallel fibers [20]. In the granule cell layer the expression corresponded to granule cells somas and dendrites [21].

We performed a multi-way ANOVA test on the expression patterns of each receptor. The test compared genetic background (C57 vs BTBR), sex (male vs female), environmental condition (standard vs enriched caging), and layer in the cerebellar cortex (molecular, Purkinje, and granular). In the case of NMDAR1/DAPI, this test showed that sex was the only significant effect ($F(\mathrm{1,32}) = 56.95, p=1.71 \times {10}^{-10}$, and the effect size was ${(\eta }^{2}= 0.44$). The same test for the expression of CB1R showed that sex (sex:$F\left(\mathrm{1,32}\right)= 11.09, p=14.00 \times {10}^{-4}, {\eta }^{2}= 0.11$) and environmental condition ($F(\mathrm{1,32}) = 16.75, p=1.00 \times {10}^{-4}, {\eta }^{2}= 0.17$) had a significant effect.

Since sex has a strong influence on the expression of both, NMDAR1 and CB1R, we performed another multi-way ANOVA separating the groups by sex. For NMDAR1 this shows that, for both males and females, the genetic background had a significant effect on the expression of this receptor (males: $F(\mathrm{1,32}) = 13.73, p=8.00 \times {10}^{-4}, {\eta }^{2}=0.22$; females: $F(\mathrm{1,32}) = 26.69, p = 1.33 \times {10}^{-5}, {\eta }^{2}=0.22$). This was also the case for the environmental condition (males: $F(\mathrm{1,32}) = 15.98, p = 3.00\times {10}^{-4}, {\eta }^{2}=0.25$; females: $F(\mathrm{1,32}) = 56.94, p = 1.66 \times {10}^{-8}, {\eta }^{2}=0.48$). We obtained the same result when performing the test for the CB1R images (Genetic background: males, $F(\mathrm{1,32})=14.91, p= 5.00\times {10}^{-4},{\eta }^{2}=0.23$; females, $F(\mathrm{1,32}) = 14.40, p = 6.00 \times {10}^{-4}, {\eta }^{2} = 0.27$; Environmental condition: males, $F(\mathrm{1,32}) = 18.21, p = 1.00 \times {10}^{-4}, {\eta }^{2} = 0.28$; females, $F\left(\mathrm{1,32}\right)= 6.27,p = 0.01, {\eta }^{2}=0.11$).

**Fig. 2:**
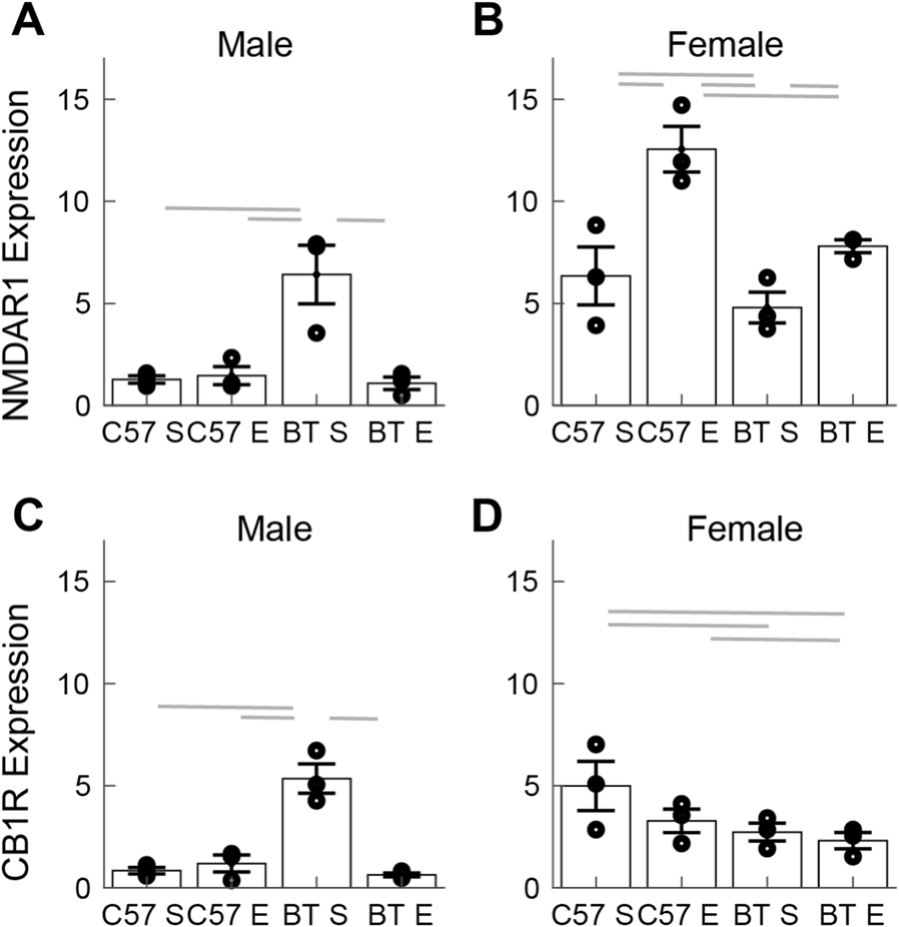
Effects of environmental enrichment on the expression of glutamate (NMDAR1) and endocannabinoid (CB1R) receptors in C57BL/6 (C57) and BTBR + Itpr3tf/J (BT) mice in the cerebellar cortex. **A** Values of NMDAR1/DAPI. The symbols correspond to the average expression of the receptor in each animal. Animal groups were from each genetic background (C57 or BT) exposed to standard (S) or enriched (E) environments. Each column shows mean and SEM. The horizontal lines indicate statistical significance (p < 0.05). **B** The same analysis as in A for female mice. **C** , **D** as in **A** and **B** for the expression of CB1R. Each data point is the average of 3 samples obtained from the same mice. We collected tissue from 3 animals for each experimental condition

Next, we performed a post-hoc multi-compare analysis of the multi-way ANOVA tests (groups separated by sex) to determine differences in the effect of environmental enrichment and genetic background in the expression of each receptor. In males, the expression of NMDAR1/DAPI in the BTBR enriched environment group was more than 5 times larger (and significantly different, mean 6.42) than for all the other experimental groups (C57 standard and enriched environment groups, and the BTBR standard environment group, Fig. 2A). The same analysis for expression of NMDAR1 in females shows that the effect of environmental enrichment is similar for the C57 and BTBR groups. In both cases, the enriched environment had a higher expression of the receptor than the corresponding standard environment group. Thus, suggesting that the genetic background does not modify the effect of environmental enrichment in females (Fig. 2B).

We also performed a post-hoc analysis for the CB1R data. This shows that the expression of CB1R in males is identical to the pattern we found for NMDAR1 males (Fig. 2C). The expression in females was different from CB1R males and from NMDAR1 males or females. The BTBR groups showed a lower ratio of CB1R/DAPI than the C57 groups. Environmental enrichment had no effect in changing the expression of CB1R (Fig. 2D).

Since we used tissue from the same animals to stain for CB1R or NMDAR1 we studied the relative expression of these receptors. We calculated the correlation coefficient between these values across all animals. We found that the correlation coefficient for all males was large and significant ($r = 0.89, p = 1.10\times {10}^{-4}$). In contrast, the correlation coefficient for females was not significant ($r = 0.10, p = 0.77$). This analysis suggests a differential regulation in the co-expression of NMDAR1 and CB1R between males and females.

## Discussion

We demonstrated that environmental enrichment affects the expression of NMDAR1s and CB1Rs in vermal lobule VII of the cerebellar cortex. Our results show differences that depended on sex and receptor type, suggesting a complex effect of environmental enrichment in receptor expression in the cerebellar cortex. These findings contribute to the use of the BTBR strain to study sexual dimorphism in neurological disorders in humans [22–24].

While there is no single environmental enrichment paradigm, in all protocols as in this study, the arena is larger than the standard cage and there is a constant reorganization of “toys” [25–29]. In our protocol, the session duration for environmental enrichment exposure was based on therapies used in human patients with ASD [30, 31]. These environmental therapies change the individual’s experience by sensorimotor stimulation, self-directed patterns of attention, and social learning [32, 33]. Our protocol reproduced these features by the daily change in textures, shapes, sizes, and location of objects, and the mouse-mouse interaction inside the arena. As such, our arena provided an enhanced sensory, motor, social, and cognitive stimulation which meets the criteria in the implementation of this paradigm [29].

Glutamate and endocannabinoid receptors and their pathways can be therapeutic targets to treat abnormal behaviors [2, 34–37]. In the BTBR mouse NMDAR1 agonist and antagonist have been used to improve sociability and spontaneous grooming [1, 38]. The activation of endocannabinoid production by acetaminophen enhances social behavior in BTBR mice [39]. BTBR animals have a mutation in the Kmo and Ext1 genes, involved in the production of a glutamate receptor antagonist and excitatory synaptic transmission [40, 41]. It is possible that the increased expression of NMDAR1 in male BTBR mice found in our work, is a compensation for the dysregulation in the function of the proteins encoded by these genes, which could have sex differences [24].

## Limitations

Our research would benefit from quantification of changes in behavior to correlate with our molecular expression measurements. A limitation in the interpretation of our results for translational applications in ASD is that while the BTBR mouse model has a strong construct validity it may not reproduce behavioral and cellular symptoms reported in human patients. In addition, data from ASD adult human postmortem brains is not available, and neither data from ASD adult after environmental enrichment, so our data cannot be contrasted.

Our study would benefit from a larger sample size. Particularly, to study small differences between the standard housing groups. However, our current results have a statistical power of 80% and the effect size shows that the magnitude of the experimental effect is medium to large [42] for the receptor expression.

We concentrated our measurements in Lobule VII because of the known effects of ASD in this area; future work should compare changes in expression across the cerebellum. As an additional limitation the two genotypes used in this work were not littermates, therefore there could be further genetic differences introduced de novo. As a future direction, other molecular techniques such as RT-qPCR should be use to quantify the NMDAR1 and CB1R expression at mRNA level.

## Supplementary Information

**Additional file 1**: **Figure S1**. Regions of interest (ROI) to calculate average fluorescent signal from a receptor marker and DAPI in the cerebellar cortex. Example image from a cerebellar Lobule VII slice expressing CB1R (green). The nuclei are marked with DAPI (blue). The image was obtained simultaneously. The molecular and granular layers were polygons drawn by hand. The Purkinje layer ROIs consisted in squares 19.8 μm on each side.

## Data Availability

All data and analysis code are available at github.com/Santamarialab/BTBR and upon request.

## Abbreviations

ASDs: Autism spectrum disorders.
BTBR: BTBR + Itpr3tf/J strain mice.
NMDR1s: N-methyl-d-aspartate receptors subunit1.
CB1Rs: Endocannabinoid receptors type1
C57: C57BL/6 strain mice group
BT: BTBR + Itpr3tf/J strain mice group
S: Standard
E: Enriched
ROIs: Regions of interest
LARC: Laboratory animal resources center
UTSA: University of texas at san antonio
PBS: Phosphate buffer saline
TBS: Tris-buffered saline
DAPI: 4′, 6-Diamidino-2-phenylindole
ANOVA: Analysis of variance
Env: Environment
Gen: Genetic background
M: Male
F: Female
P: Postnatal day
