# Supplementary material for: Effects of environmental enrichment and sexual dimorphism on the expression of cerebellar receptors in C57BL/6 and BTBR + Itpr3tf/J mice
Source: BMC Res Notes. 2022 May 13;15:175. doi: 10.1186/s13104-022-06062-8 (PMC9103090; doi:10.1186/s13104-022-06062-8)
Supplement: Supplementary file 1 — Additional file 1: Figure S1. Regions of interest (ROI) to calculate average fluorescent signal from a receptor marker and DAPI in the cerebellar cortex. Example image from a cerebellar Lobule VII slice expressing CB1R (green). The nuclei are marked with DAPI (blue). The image was obtained simultaneously. The molecular and granular layers were polygons drawn by hand. The Purkinje layer ROIs consisted in squares 19.8 μm on each side. [file 13104_2022_6062_MOESM1_ESM.pdf]

Supplementary Materials

Effects of environmental enrichment and sexual dimorphism on the expression of cerebellar receptors in C57BL/6 and BTBR +Itpr3tf/J mice

Daniela Monje-Reyna[1], Jorge Manzo Denes[2], Fidel Santamaria[1*]

1 Department of Biology, University of Texas at San Antonio, San Antonio, TX 78249

2 Brain Research Center, Veracruzana University, Xalapa, Veracruz, Mexico

* Corresponding author: Fidel.santamaria@utsa.edu

Immunofluorescence

We anesthetized mice with 4 mL isoflurane in an evaporation chamber and kept them in deep anesthesia using the nose cone method. Animals were transcardially perfused with saline followed by 4% paraformaldehyde (0.2 M phosphate buffer, pH 7.4). The brains were removed and immersed in the same fixative for 48 h in sucrose solution (30%). Using a cryostat (Leica CM1510 S), we obtained 45 µm thick sagittal sections from frozen cerebellum that contained lobule VII. We performed a free-floating immunohistochemical process to stain for NMDAR1 and CB1Rs, using the following procedure in all the cases. All tissues were processed at the same time.

To label NMDAR1 we rinsed the slices with PBS 0.1 % Tween 20 (PBS-T) 3 times for 10 minutes shaken at 170 rpm (Compact Digital MicroPlate Shaker, Thermo Fisher Scientific). Then we blocked the slices with 5 % goat serum (Thermo Fisher Scientific) PBS-T for 1 hour and incubated them with the same solution containing a 1:250 dilution of NMDAR1 rabbit polyclonal antibody (RRID: AB_2112003), shaking overnight at 4°C. Sections were washed with PBS-T as before and incubated with goat anti-rabbit IgG linked to Alexa-647 (RRID: AB_2535813) at a 1:1500 dilution for 50 minutes, protected from light. After this time, the sections were washed again 3 times. All antibodies from Thermo Fisher Scientific.

To label CB1R, we rinsed the slices with TBS and blocked the slices with 10 % donkey serum (Jackson Immuno Research Laboratories, RRID: AB_2337258) and TBS containing 0.3 % Triton-X100 (TBS-T) for 1.5 hours. Sections were then incubated with TBS-T 5 % donkey serum and a 1:500 dilution of CB1R guinea pig polyclonal antibody (Synaptyc Systems, RRID: AB_2661870), shaken overnight at 4°C. Sections were washed with TBS as before and incubated in a secondary antibody linked to Cy3 anti-guinea pig IgG (FluoTag-X2, NanoTag Biotechnologies Synaptyc Systems, RRID: AB_2744577) at a dilution 1:500 for 1 hour, protected from light. Finally, the sections were washed 3 times as before. In all cases, we stained the nuclei with DAPI (4′,6-diamidino-2-phenylindole, Thermo Fisher Scientific) at a 1:500 dilution for 2 minutes and we mounted the tissue with ProLong Diamond Antifade Mountant (Thermo Fisher Scientific).

Fluorescence microscopy

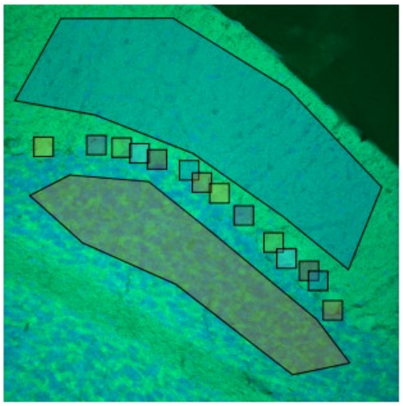

Figure S1. Regions of interest (ROI) to calculate average fluorescent signal from a receptor marker and DAPI in the cerebellar cortex. Example image from a cerebellar Lobule VII slice expressing CB1R (green). The nuclei are marked with DAPI (blue). The image was obtained simultaneously. The molecular and granular layers were polygons drawn by hand. The Purkinje layer ROIs consisted in squares 19.8 μm on each side.
